# Association of the healthy eating index with overweight and obesity among children aged 4 to 9 years in the United Arab Emirates: a cross-sectional study

**DOI:** 10.3389/fpubh.2025.1644113

**Published:** 2025-10-31

**Authors:** Leila Cheikh Ismail, Ayesha S. Al Dhaheri, Nada Abbas, Katia AbuShihab, Fatima Al Zahraa Chokor, Lynda O’Neill, Habiba Ali, Maysm N. Mohamad, Nahla Hwalla, Lara Nasreddine, Farah Naja

**Affiliations:** ^1^Department of Clinical Nutrition and Dietetics, College of Health Sciences, University of Sharjah, Sharjah, United Arab Emirates; ^2^Nuffield Department of Women’s & Reproductive Health, University of Oxford, Oxford, United Kingdom; ^3^Department of Nutrition and Health, College of Medicine and Health Sciences, United Arab Emirates University, Al Ain, United Arab Emirates; ^4^Department of Public Health, College of Health Sciences, QU Health, Qatar University, Doha, Qatar; ^5^Nestlé Institute of Health Sciences (NIHS), Route du Jorat, Switzerland; ^6^Nutrition and Food Sciences Department, Faculty of Agriculture and Food Sciences, American University of Beirut, Beirut, Lebanon

**Keywords:** children, diet quality, healthy eating index, pediatric obesity, United Arab Emirates

## Abstract

**Background:**

Pediatric obesity is a growing public health concern globally and in the United Arab Emirates (UAE). Understanding diet quality in relation to obesity risk is essential for developing effective interventions. The main objective of this study is to evaluate dietary quality, as measured by the Healthy Eating Index (HEI), and examine its association with overweight and obesity among children aged 4 to 9 years in the UAE.

**Methods:**

Data for this study were derived from a representative survey conducted in the three largest Emirates of the UAE: Abu Dhabi, Dubai, and Sharjah. A total of 426 children aged 4 to 9 years, recruited using a stratified cluster sampling frame, were included in the analysis. Data collection was conducted through face-to-face interviews with the main caregiver. Dietary intake was assessed via a 24-h recall. The HEI was used to examine dietary quality. Anthropometric data were collected to classify weight status using WHO BMI-for-age z-scores. Simple and multiple logistic regression models assessed associations between HEI scores and overweight/obesity.

**Results:**

Only 9.4% of children achieved a Moderate to Good HEI score (≥60), while 90.6% fell into the Poor category. Children with higher HEI scores had significantly lower odds of being overweight or obese (adjusted OR = 0.32, 95% CI: 0.13–0.79, *p* = 0.014). Key dietary gaps were identified in vegetable, whole grain, and seafood/plant protein intake. Determinants of better HEI scores included higher paternal education, while maternal employment was associated with poorer diet quality.

**Conclusion:**

Diet quality among children in the UAE is generally suboptimal and is significantly associated with overweight and obesity. The HEI is a valuable tool for identifying dietary gaps and informing targeted nutritional interventions to reduce obesity risk in this population.

## 1 Introduction

Pediatric obesity has become a major global health concern, with its prevalence rising to unprecedented levels among children and adolescents worldwide (1). In 1990, pediatric overweight and obesity affected 8% of children and adolescents. This rate has been steadily increasing, reaching over 20% of children and adolescents being overweight or obese in 2022 (2). Global data showed a 1.5-fold increase in the prevalence of pediatric obesity during the period from 2012 to 2023 compared to 2000 to 2011 (3). Alarmingly, projections indicate that by 2030, up to 30% of children and adolescents worldwide may be affected (4).

Such a rise in pediatric obesity is a worldwide health concern, due to its short-term and long-term consequences on physical and mental well-being (3, 4). A vast range of short-term physical comorbidities associated with pediatric obesity have been observed in a plethora of literature (5–12). For instance, pediatric obesity is associated with children developing hypertension (6), dyslipidemia (7), fatty liver disease (8), metabolic syndrome (9), diabetes (10), asthma (11), sleep apnea (12), postural malalignment (13), and other pathologies. Mental health has also been affected in children with excess weight. A meta-analysis has shown that children with overweight or obesity have higher risks of anxiety and poor self-esteem (14). Obesity during childhood may also have long-lasting consequences. Children with obesity were almost 3 times more likely to develop diabetes (15), and 4 times more likely to develop major depressive disorder as adults, compared to children without (16). As such, the adverse short- and long-term health consequences of pediatric obesity and its heavy burden on public health necessitate developing preventative interventions and programs addressing its determining and modulating factors.

Obesity is a multifactorial disease, with its core etiology rooted in poor diet quality and a chronic excess of energy intake over expenditure (17–20). Numerous studies have explored the role of individual nutrients in obesity, such as protein—where lower intake has been associated with higher energy consumption—and micronutrients like vitamins A, B1, B2, B12, and D, which show inverse associations with obesity risk (21). While these studies contributed significantly to a better understanding of the diet-disease association, focusing solely on single nutrients may oversimplify the intricate relationship between diet and obesity risk. A more holistic evaluation—one that considers overall dietary patterns—offers a broader and more meaningful understanding. A systematic review assessing the association between pediatric obesity and various dietary patterns across several countries reported that unhealthy diets—typically characterized by high intake of sugar sweetened beverages, fast foods, refined grains, and others—increased the risk of children becoming overweight or obese (22). Conversely, healthy dietary patterns rich in fruits, vegetables, whole grains, fish, nuts, legumes, and yogurt were linked to a lower likelihood of obesity (22). To support this broader more holistic approach to examining the association between diet and obesity, tools like the HEI have become increasingly valuable (23). Developed in 1995 (24), HEI is one of the most frequently utilized diet quality scoring tools worldwide (25), with consistently documented validity and reliability (26). By assessing overall diet quality in relation to established dietary guidelines, the HEI captures both the adequacy and moderation aspects of dietary intake.

The United Arab Emirates (UAE), a member of the Gulf Cooperation Council (GCC), is known for its rapid economic growth and urban expansion. Supported by well-developed infrastructure and advanced technological services, the country offers a modern lifestyle marked by convenience and accessibility. As in many highly urbanized settings, this way of life often includes easy access to a wide variety of food options and reduced opportunities or need for physical activity—factors that contribute to increased risks of excessive weight gain and obesity (27). As a result, the UAE is currently experiencing a high burden of obesity and its comorbidities, among both adults and children (28, 29). The World Obesity Federation (30) reports a high prevalence of obesity among children of the UAE, with one cross-sectional study identifying a prevalence of overweight or obesity among 40% of 11–14-year-old Emirati children (31). These rates of pediatric obesity in the UAE call for prompt dietary and public health interventions. Nonetheless, developing such interventions first requires a comprehensive evaluation of the general eating patterns and the specific dietary components that are most relevant to obesity among children in the UAE. The main aim of this is to examine overall diet quality using HEI and investigate the association between HEI scores with overweight and obesity among children aged 4 to 9 years in the UAE. Additionally, it aims to assess the level of adherence to the specific dietary components measured by the HEI, highlighting gaps and potential areas for dietary intervention in this population.

## 2 Methods

### 2.1 Study design and subjects’ selection

The data for this study were derived from a previously conducted large-scale cross-sectional survey designed to assess food consumption patterns, dietary intake, and nutrient sources among children between 4 and 12.9 years of age in the UAE. The original survey adhered to the protocol of the Global Kids Nutrition and Health Study (32). In the UAE, the KNHS was implemented in the three largest Emirates: Abu Dhabi, Dubai, and Sharjah. Children were recruited using a stratified random cluster sampling approach, where the Emirates formed the strata. Schools, including preschools, were treated as clusters and randomly selected within each stratum. At the selected schools, all children who met the established inclusion criteria were invited to participate in the study (aged between 4 and 12.9 years and free from any medical conditions that might alter their dietary intakes or anthropometric measurements, such as inborn errors of metabolism, chronic medical conditions, or physical disabilities). Children of Arab non-nationals who had resided in the UAE for less than 3 years, as well as those whose mothers were younger than 18 years of age, were excluded from participation. Further information regarding the specific sampling methods employed in the UAE KNHS is documented in greater detail elsewhere (33). To enable comparative analyses between nationals and non-nationals, a sample of Arab non-national children was also included, with a recruitment ratio of 2:1 (nationals to non-nationals). The original survey included a total sample of 646 children, consisting of 431 national participants and 215 non-national participants. For this study, data for pre-adolescent children aged between 4 and 9 years were used (*n* = 426) to minimize confounding by pubertal growth and maturation.

The ethical review bodies that have examined and approved the study included: the Institutional Review Board (IRB) of the American University of Beirut (AUB), the IRB of the United Arab Emirates University (UAEU), Dubai Health Authority (DHA), UAE Ministry of Health and Prevention (MOHAP), Ministry of Education in the United Arab Emirates (MOE), and the University of Sharjah (UOS). Participating families were offered a $15 book voucher as a token of appreciation for their time spent completing the surveys and the various assessments conducted in the study. In this study, a post-hoc power analysis was conducted using G*Power 3.1 to ensure that the sample size of 426 participants provided the needed statistical power for the analyses. With a two-tailed test, a significance level (*α*) of 0.05, and a desired power (1-*β*) of 0.80, the available sample size allowed the detection of an odds ratio of 0.6 for the association between the HEI and obesity (overweight/obese vs. normal weight) (34).

### 2.2 Study protocol and data collection

A detailed description of the study protocol and the data collection is found elsewhere (33). Briefly, between June 2019 and March 2020, trained research nutritionists visited the selected preschools and schools. Parents agreeing to participate in this study were interviewed, after signing a written consent form. The interview lasted for around 30 min and took place in a private room at the school’s premises. The main caregiver and the child were both present during the interview. In this study, all caregivers were the mothers of the children. The data collection included a multicomponent questionnaire in addition to anthropometric assessments of both the mother and the child. The questionnaire consisted of three main sections. The first section included questions about the socio-demographic characteristics. The second section addressed the lifestyle habits of the child such as eating while watching TV, fast food consumption frequency, and physical activity. The Youth Physical Activity Questionnaire (Y-PAQ) was used to obtain the number of hours of physical activity during the weekdays and weekends (35).

The third section of the questionnaire addressed the dietary intake of the child and included the multiple-pass 24-h recall (24-HR) method, developed by the USDA. Mothers, as proxies, relayed the information in the 24-HR. The standard five-step process used in completing the 24-HR helps to minimize recall bias and enhance accuracy. The first step gathered a general overview of the child’s meals and snacks from the previous 24 h. In the second step, more detailed information on food types, preparation methods, and condiments was collected. The third step involved portion size estimation using household measures and visual charts to improve accuracy. Dietary data were analyzed using Nutritionist Pro Software, with local recipes and mixed dishes from the UAE entered individually. The analysis used several food composition databases, including the USDA database and Middle East-specific tables.

Standardized protocols and equipment were used to obtain the weight and height of the mother and the child. The Body Mass Index (BMI) of mothers was calculated as the ratio of weight (Kg)/height (m)^2^. Children’s weight status was classified as wasted, normal, at risk of overweight/overweight, or obese using the age-specific cutoffs of the BMI for Age Z-Score (BAZ), as per the WHO Child Growth Criteria (36).

### 2.3 Derivation of the healthy eating index

In this study, the HEI was calculated as described in Krebs-Smith et al. (37), using the dietary intake data obtained by the 24HR. The HEI consisted of 13 components categorized into two groups: adequacy components and moderation components. Adequacy components assessed whether individuals are consuming sufficient amounts of foods recommended by dietary guidelines, such as fruits, vegetables, whole grains, dairy products, fatty acid ratio (PUFAs+MUFAs)/SFAs, and protein foods. On the other hand, moderation components evaluate the intake of food groups that should be consumed in limited quantities, including refined grains, sodium, added sugars, and saturated fats. Each of the 13 components is scored based on intake, with higher scores allocated for higher intakes of adequacy components and lower intakes of moderation components. Scores for each component range from 0 to 5 or 0 to 10, depending on the specific component. Intakes between the minimum and maximum standards are scored proportionately. The criteria for obtaining the maximum and minimum scores for each component are detailed in Table 1. The individual component scores are then summed to provide a total HEI score ranging from 0 to 100. A score closer to 100 indicates a healthier diet that aligns well with dietary guidelines, while lower scores suggest areas where dietary improvements are needed.

**Table 1:** Distribution of the study population across the maximum and minimum scores of each of the HEI (*n* = 426).

Component	Maximum points	Standard for maximum score^a^	n (%) meeting maximum score	Standard for minimum score of zero	n (%) meeting minimum score
Adequacy components		per 1,000 kcal			
Total Fruits^b^	5	≥ 0.8 cup equiv.,	166 (38.7%)	No Fruit	137 (31.9%)
Whole Fruits^c^	5	≥ 0.4 cup equiv.	151 (35.2%)	No Whole Fruit	221 (51.5%)
Total Vegetables^d^	5	≥ 1.1 cup equiv.	3 (0.7%)	No Vegetables	249 (58%)
Greens and Beans	5	≥ 0.2 cup equiv.	5 (1.2%)	No Dark Green Vegetables or Legumes	419 (97.7%)
Whole Grains	10	≥ 1.5 oz. equiv.	40 (9.3%)	No Whole Grains	314 (73.2%)
Dairy^e^	10	≥ 1.3 cup equiv.	56 (13.1%)	No Dairy	47 (11%)
Total Protein Foods^d^	5	≥ 2.5 oz. equiv.	158 (36.8%)	No Protein Foods	104 (24.2%)
Seafood and Plant Proteins^f^	5	≥ 0.8 oz. equiv.	41 (9.6%)	No Seafood or Plant Proteins	362 (84.4%)
Fatty Acids^g^	10	(PUFAs^h^ + MUFAs^i^) / SFAs^j^ ≥ 2.5	77 (17.9%)	(PUFAs^h^ + MUFAs^i^) / SFAs^j^ ≤ 1.2	134 (31.2%)
Moderation components					
Refined Grains	10	≤ 1.8 oz. equiv.	126 (29.4%)	≥ 4.3 oz. equiv. per 1,000 kcal	175 (40.8%)
Sodium	10	≤ 1.1 grams	207 (48.3%)	≥ 2.0 grams per 1,000 kcal	10 (2.3%)
Added Sugars	10	< 6.5% of energy	120 (28%)	≥ 26% of energy	194 (45.2%)
Saturated Fats	10	≤ 8% of energy	124 (28.9%)	≥ 16% of energy	29 (6.8%)

^a^Intakes between the minimum and maximum standards are scored proportionately comp. Component scores are summed to create the total score. ^b^Includes 100% fruit juice. ^c^Includes all forms except juice. ^d^Includes beans, peas, and lentils. ^e^Includes all milk products, such as fluid milk, yogurt, and cheese, and fortified soy beverages. ^f^Includes seafood, nuts, seeds, soy products (other than beverages), and beans, peas, and lentils. ^g^Ratio of poly- and monounsaturated fatty acids (PUFAs and MUFAs) to saturated fatty acids (SFAs). ^h^PUFAs, polyunsaturated fatty acids. ^i^MUFAS, monounsaturated fatty acids. ^j^SFAs, saturated fatty acids.

We applied the HEI-2015 scoring algorithm, organized into adequacy and moderation components and expressed per 1,000 kcal, to children aged 4–9 years. The density-based nature of HEI-2015 allows comparisons across ages with different energy requirements and has been used in multiple pediatric studies (38–41). Accordingly, we used the standard HEI-2015 minimum and maximum standards for each component and summed components to obtain the total score (0–100), without age-specific rescaling.

### 2.4 Statistical analysis

All statistical analyses were conducted using Stata version 15. Descriptive statistics were used to summarize the baseline characteristics of the study population, with continuous variables expressed as means and standard deviations (Mean ± SD) and categorical variables as frequencies and percentages. Comparisons between the wasted/normal BAZ group and the overweight/obese BAZ group were performed using independent sample t-tests for continuous variables and chi-square tests for categorical variables. The HEI scores were categorized as “Poor” or “Moderate to Good” based on Bloom cutoffs: Poor (0–60) and Moderate/Good (60–100). The percent contribution of each HEI component to the total HEI score was also reported. Multivariate logistic regression models were used to assess the associations between HEI and overweight/obesity, adjusting for potential confounders identified in the bivariate analysis between weight status and baseline characteristics (variables with *p*-value <0.2 were included in the adjusted model). Crude and adjusted odds ratios (aOR) with 95% confidence intervals (CI) were reported for these associations. Simple and multiple logistic regression models were employed to identify determinants for moderate to good vs. poor HEI, with results presented as crude and adjusted odds ratios. Variables with *p*-values <0.2 in the univariate analysis were included in the multivariable model. All analyses were conducted with a two-sided alpha level of 0.05 for statistical significance.

## 3 Results

The baseline characteristics of the study population are summarized in Table 2. The study population included 426 children, with 377 classified as having a wasted/normal BMI-for-age z-score (BAZ), and 49 classified as overweight/obese. The mean age of the children was 6.18 ± 1.40 years, while the mean age of their mothers was 35.82 ± 6.09 years. The mean mother’s BMI was 28.12 ± 5.08 kg/m^2^. The sample was nearly evenly split by child sex, with 47.9% male and 52.1% female participants. The majority of the children were Emirati (64.3%). Regarding parental employment and education, 51.4% of the mothers had up to a high school education, and 46.2% of the mothers were employed. Fathers were predominantly employed (93.6%), with 48.6% having up to a high school education. About 35% of families reported earning up to 30,000 DHS, 18.8% earning above 30,000 DHS, and 46.2% were unsure or unwilling to disclose their income. Regarding lifestyle factors, 65% of children reported eating while watching TV, and 65.8% consumed fast food at least once per week. Breakfast consumption was reported by almost all children (97.7%). The average time spent on physical activity during weekdays was 5.71 ± 5.61 and during weekends was 1.36 ± 1.99 h. Only the mother’s BMI and the number of physical activity hours during weekdays showed significant differences between the groups based on the child’s BAZ. Mothers of overweight/obese children had a higher mean BMI (31.74 vs. 27.66 kg/m^2^, *p* = 0.016). Additionally, children in the overweight/obese group had fewer mean physical activity hours during weekdays (4.11 ± 3.46) compared to normal-weight children (5.91 ± 5.80, *p* < 0.001).

**Table 2:** Baseline characteristics of the study population (*n* = 426).

Characteristics	Total (*n* = 426)		Wasted/normal BAZ^*^ (*n* = 377)		Overweight / obese BAZ^*^ (*n* = 49)		*p*-value
	n	%	n	%	n	%	
Child’s Age (years; Mean ±SD)	6.18	1.40	6.13	1.39	6.56	1.42	0.849
Mother’s Age (years; Mean ±SD)	35.82	6.09	35.61	6.17	37.50	5.11	0.126
Mother’s BMI (kg/m^2^; Mean ±SD)	28.12	5.08	27.66	4.73	31.74	6.19	**0.016**
Child’s sex							
Male	204	47.9	177	46.9	27	55.1	0.283
Female	222	52.1	200	53.1	22	44.9	
Nationality							
Emirati	274	64.3	246	65.3	28	57.1	0.265
Non-Emirati	152	35.7	131	34.7	21	42.9	
Mother’s education							
Up to high school	219	51.4	196	52	23	46.9	0.797
University degree	190	44.6	166	44	24	49	
Graduate / professional degree	17	4	15	4	2	4.1	
Mother’s employment status							
Unemployed	229	53.8	207	54.9	22	44.9	0.186
Employed	197	46.2	170	45.1	27	55.1	
Father’s education							
Up to high school	205	48.6	176	47.2	29	59.2	0.072
University degree	188	44.5	168	45	20	40.8	
Graduate / professional degree	29	6.9	29	7.8	0	0	
Father’s employment status							
Unemployed	27	6.4	23	6.2	4	8.2	0.591
Employed	395	93.6	350	93.8	45	91.8	
Family income							
Up to 30,000 DHS	149	35	133	35.3	16	32.7	0.765
30,000 DHS and above	80	18.8	72	19.1	8	16.3	
Do not know/Refuse	197	46.2	172	45.6	25	51	
Eating while watching TV							
No	149	35	127	33.7	22	44.9	0.122
Yes	277	65	250	66.3	27	55.1	
Fast food frequency							
Less than once per week	144	34.2	129	34.7	15	30.6	0.224
Once per week	188	44.7	169	45.4	19	38.8	
More than once per week	89	21.1	74	19.9	15	30.6	
Breakfast consumption^**^							
No	10	2.3	9	2.4	1	2	0.880
Yes	416	97.7	368	97.6	48	98	
Physical activity during the weekdays (hours) (Mean ±SD)	5.71	5.61	5.91	5.8	4.11	3.46	**<0.001**
Physical activity during the weekend (hours) (Mean ±SD)	1.82	2.37	1.85	2.40	1.61	2.19	0.411

*BAZ, BMI-for-age z-score. Defined as follows: BAZ ≤ +2 indicates wasting/normal, and +2 < BAZ ≤ +3 indicates overweight/obese (World Health Organization, Department of Nutrition for Health and Development. WHO Child Growth Standards: Length/Height-for-Age, Weight-for-Age, Weight-for-Length, Weight-for-Height and BMI-for-Age: Methods and Development. Geneva, Switzerland: WHO Press; 2006). **Information about breakfast consumption was derived from the 24-HR. Bold values indicate statistically significant differences between groups.

Table 1 shows the distribution of HEI component scores. For adequacy, 38.7 and 35.2% of participants reached the maximum for total and whole fruits, respectively. Only 0.7% scored the maximum for total vegetables, and 58% scored the minimum. A notable 97.7% had minimal greens and beans intake, and 73.2% scored the minimum for whole grains. For dairy, 13.1% reached the maximum, while 36.8% met protein intake targets. Seafood and plant proteins had low adherence, with 84.4% scoring the minimum. Regarding fatty acids, 17.9% met the maximum score, while 31.2% scored the minimum. For moderation components, 29.4% achieved the maximum score for refined grains, and 48.3% for sodium. Added sugars and saturated fats had 28 and 28.9% meeting the maximum score, respectively, with 45.2% scoring the minimum for sugars.

**Figure 1:**
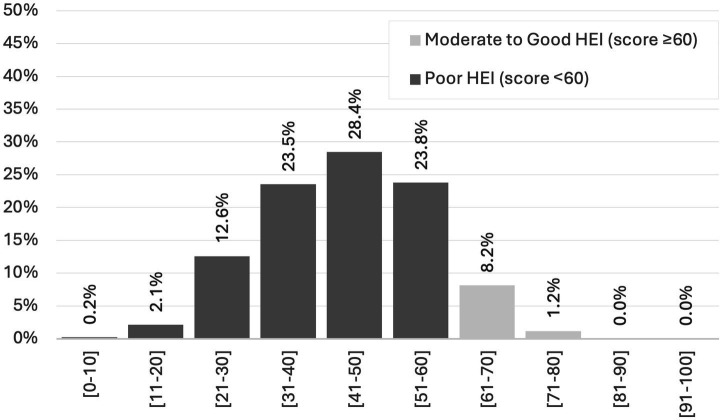
Distribution of the HEI total scores (*n* = 426).

The distribution of HEI total scores is presented in Figure 1. The majority of scores fall within the ranges of 31–40 (23.5%), 41–50 (28.4%), and 51–60 (23.8%). Scores of 60 or higher, indicating Moderate to Good HEI, account for 9.4% of the total, while scores of 60 or lower, indicating Poor HEI, make up the remaining 90.6%.

**Figure 2:**
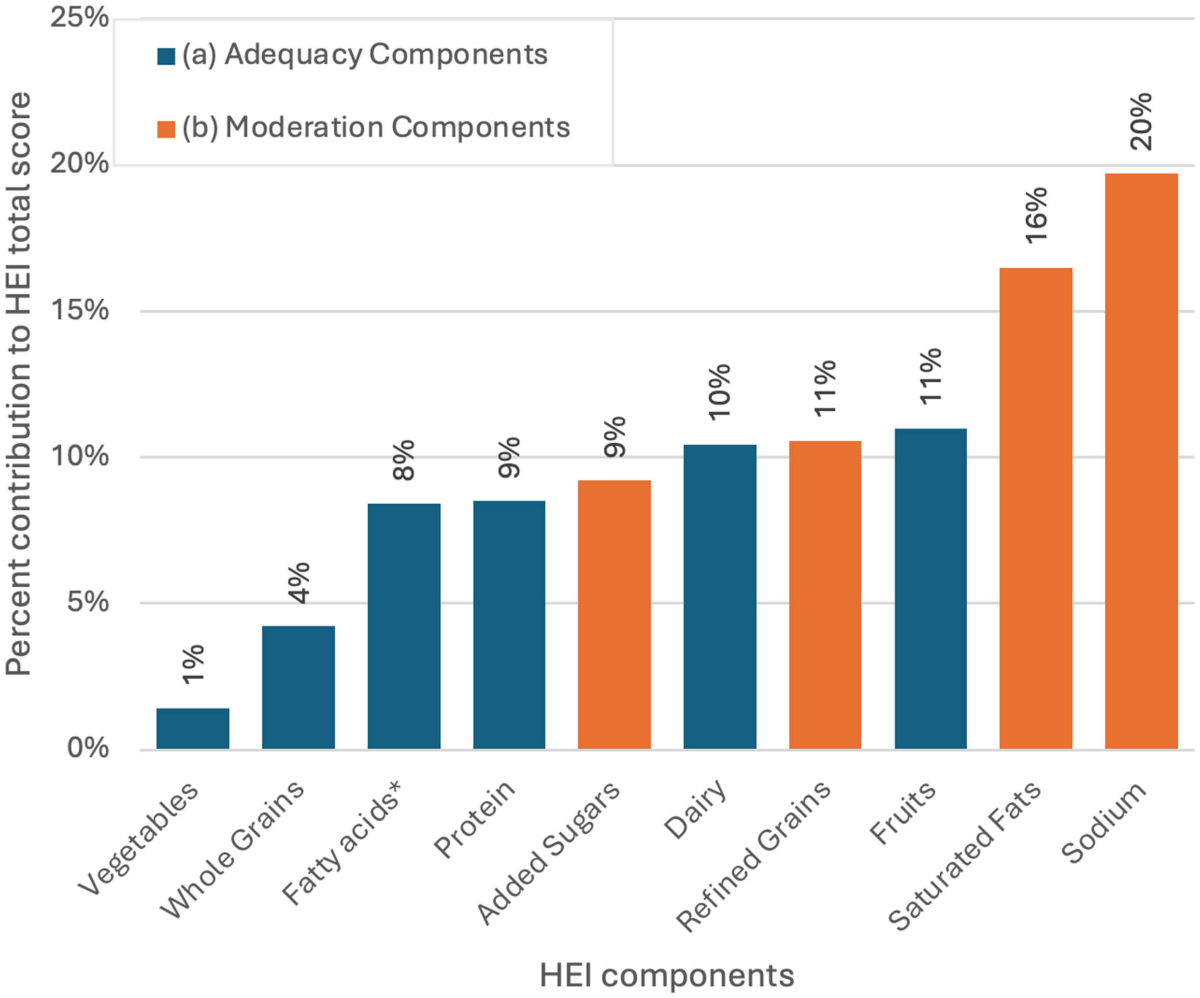
Percent contribution of each of the HEI components to the total HEI score (*n* = 426). * (PUFAs+MUFAs)/SFAs. **(A)** Adequacy components include foods that should be consumed in sufficient quantities to meet nutrient needs and promote overall good health. A higher intake results in a higher score. **(B)** Moderation components include dietary components that should be limited or consumed in small amounts. A lower intake results in a higher score.

Figure 2 presents the percentage contribution of various components to the overall HEI score, divided into adequacy and moderation components. Adequacy components measured include fruit (11%), dairy (10%), protein (9%), and fatty acids [(PUFAs + MUFAs)/SFAs] (9%) as the highest contributors. Whole grains (4%) and vegetables (1%) show lower contributions, suggesting insufficient consumption. Moderation components consist of sodium (20%) and saturated fats (16%) as the highest contributors, indicating these are relatively well-moderated within the diet, contributing positively to the HEI score. In contrast, lower contributions from Refined Grains (11%) and Added Sugars (9%) suggest less moderation, resulting in a lower positive contribution to the HEI.

Table 3 shows the associations of HEI with children’s weight status. Children with moderate to good HEI scores were less likely to be overweight or obese compared to those with poor HEI scores (aOR = 0.32, 95% CI: 0.13–0.79, *p* = 0.014).

**Table 3:** Predictors of overweight and obesity by HEI in the study population (*n* = 426).

HEI category	Overweight/Obesity BAZ			
	OR (95% CI)	*p*-value	aORˠ (95% CI)	*p*-value
Poor HEI (reference)	1			
Moderate to good HEI	0.45 (0.23–0.90)	0.024*	0.32 (0.13–0.79)	0.014*

ˠaOR, Odds Ratio adjusted for child’s age and sex, mother’s age, mother’s BMI, mother’s employment status, father’s education, crowding index, eating while watching TV, and physical activity hours. Significant at *p* < 0.05.

Results of the multiple logistic regression examining the determinants of moderate to good HEI Scores include the mother’s employment status, father’s education, fast food frequency, and physical activity (Table 4). Employed mothers were less likely to have children with moderate to good HEI scores (aOR = 0.65, 95% CI: 0.43–1.00, *p* = 0.049). Fathers with a graduate or professional degree were more than twice as likely to have children with moderate to good HEI scores compared to those up to high school education (aOR = 2.69, 95% CI: 1.12–6.39, *p* = 0.027). Breakfast consumption increases the odds of a good HEI by over seven-fold, but this result did not reach statistical significance (aOR = 7.59, 95% CI: 0.93–61.8, *p* = 0.066). (Data not shown).

**Table 4:** Predictors of the HEI score by sociodemographic characteristics, as derived from simple and multiple logistic regression analyses* (*n* = 426).

Sociodemographic characteristics	Moderate-to-good HEI score							
	OR	*p*-value	95% CI		aORˠ	*p*-value	95% CI	
Child’s Age (years)	0.98	0.806	0.86	1.13	1.05	0.512	0.90	1.23
Mother’s Age (years)	1	0.835	0.96	1.03	0.99	0.687	0.96	1.03
Mother’s BMI (kg/m^2^)	1.01	0.535	0.97	1.06				
Child’s sex								
Male	1				1			
Female	0.9	0.585	0.61	1.32	0.87	0.499	0.57	1.32
Nationality								
Emirati	1							
Non-Emirati	1.17	0.445	0.78	1.75				
Mother’s education (overall *p*-value 0.258)								
Up to high school	1							
University degree	1.32	0.168	0.89	1.97				
Graduate / professional degree	0.71	0.534	0.24	2.09				
Mother’s employment status								
Unemployed	1				1			
Employed	0.72	0.106	0.49	1.07	**0.64**	**0.037**	**0.41**	**0.97**
Father’s education (overall *p*-value 0.012)								
Up to high school	1				1			
University degree	0.85	0.435	0.57	1.28	0.77	0.250	0.50	1.20
Graduate / professional degree	**2.85**	**0.012**	**1.26**	**6.44**	**2.74**	**0.024**	**1.14**	**6.56**
Father’s employment status								
Unemployed	1							
Employed	1.63	0.258	0.7	3.82				
Family income (overall *p*-value 0.978)								
Up to 30,000 DHS	1							
30,000 DHS and above	0.97	0.9	0.55	1.68				
Do not know/Refuse	1.02	0.924	0.66	1.58				
Eating while watching TV								
No	1							
Yes	1.08	0.704	0.72	1.62				
Fast food frequency (overall *p*-value 0.235)								
Less than once per week	1							
Once per week	0.71	0.129	0.46	1.1				
More than once per week	0.68	0.168	0.4	1.17				
Breakfast consumption								
No	1				1			
Yes	6.1	0.088	0.77	48.57	7.10	0.066	0.88	57.50
Physical activity during the weekdays (hours)	1.01	0.473	0.98	1.05				
Physical activity during the weekend (hours)	1.1	0.059	1	1.21	0.16	0.143	0.01	1.86

*In the regression analyses the dependent variable was HEI scores grouped as Poor or Moderate / Good HEI. ˠaOR: Odds Ratio adjusted for variables with an overall *p*-value <0.2 in the simple regression model. Values in bold are statistically significant.

## 4 Discussion

This study is among the first to investigate the association between overall diet quality, as measured by the HEI, and the prevalence of overweight and obesity among young children aged 4 to 9 years in the UAE. The findings revealed suboptimal dietary quality across the study population, with over 90% of children falling into the “Poor” HEI category. Importantly, higher HEI scores were inversely associated with the likelihood of overweight and obesity, even after adjusting for potential confounders. In a region that faces significant challenges caused by chronic non-communicable diseases this understanding of the diet quality of its younger people is critically important to shaping a public health response.

In the present study, lower HEI scores were significantly associated with increased odds of overweight and obesity, reinforcing the growing international evidence that overall diet quality plays a critical role in childhood obesity prevention. This finding is consistent with recent results from the TX Sprouts trial in the United States, which demonstrated that improvements in HEI-2020 scores were associated with reductions in body fat percentage among children, primarily driven by higher whole grain and lower refined grain intake; however, no associations were found with BMI or waist circumference (40). Similarly, analysis of the National Health and Nutrition Examination Survey (2009–2014) by Thomson et al. (2021) showed that HEI-2015 scores were consistently low across all BMI categories in U. S. children, with no significant differences by weight status but marked disparities by age and race/ethnicity (42). In Kuwait, Al-Farhan et al. (2024) found that none of the participating schoolchildren achieved a “good” HEI score, and although poor diet quality was inversely associated with obesity in unadjusted models, this association weakened after accounting for confounders (39). Supporting these observations, a large-scale NHANES-based study by Zheng et al. (2023) reported that higher HEI-2015 scores were associated with a reduced risk of overweight among children, though not significantly linked to obesity risk after full adjustment, underscoring the nuanced nature of diet–weight associations in pediatric populations (41). Further longitudinal evidence from the EPOCH study in Colorado revealed that among girls, each 10-point increase in HEI-2010 score at age 10 was associated with a significant reduction in BMI trajectory over the transition to adolescence, while no such associations were found among boys (43). Interestingly, findings from Iran by Askari et al. challenge the expected direction of association indicating that children in the highest tertile of HEI-2015 scores were more likely to be overweight, while no significant associations were observed between diet quality and other anthropometric indicators (38). Therefore, these findings highlight the context-specific nature of the relationship between diet quality and anthropometric outcomes in childhood, suggesting that while the HEI serves as a valuable indicator of obesity risk, its interpretation should be informed by factors such as age, sex, and culturally specific dietary patterns.

In addition, parental education was associated with a better HEI, reflecting the major impacts that access and health literacy have on diet. These socioeconomic factors often intersect with parental weight status and household dietary behaviors (44, 45). For example, maternal BMI has been shown to correlate with children’s weight status and their diet quality, likely due to shared home food environments, meal patterns, and modeling of eating behaviors (46). Given the high incidence of cardiometabolic disease in the UAE, the high rates of poor HEI scores among its young people, and the associations between it and overweight and obesity, are worthy of significant note, and action. Family-based interventions that address nutrition within the broader household context are particularly relevant, as evidence suggests they can achieve more sustainable improvements in children’s diet quality and weight outcomes (47).

Our findings showed that children of employed mothers were significantly less likely to achieve moderate to good HEI scores. This finding aligns with research from Egypt suggesting that maternal employment may cause time constraints impacting food preparation at home (48). Similarly, Ferdous et al. indicated that maternal employment was associated with undernutrition in children (49). In the UAE, dietary transitions and dual-income households are increasingly common, highlighting the need for supportive policies that enable working parents to maintain healthy food environments at home, including access to affordable, nutritious food and more flexible work arrangements.

In the last two decades, there have been significant changes to the cultural landscape of the UAE, particularly in its urban centers (27). This has caused a move away from traditional food and eating practices toward a more stereotypical Western diet, with a rapid increase in consumption of foods high in added sugars, saturated fats, and sodium, particularly in younger people (50). These changes to key components of lifestyle medicine have led to significant increases in diet-related chronic diseases, with the UAE having significant rates of cardiometabolic diseases, and overweight and obesity (29). Many of these chronic diseases find their origins in early life, making childhood and adolescence a key window for effective interventions. In this study, we found widespread low diet quality across the UAE. The rates of children and adolescents with scores suggesting ‘poor’ diet quality in the UAE were higher than other studies in Middle Eastern countries. For example, a study in Turkey found that 42.8% of children had HEI scores lower than 50 (51), compared to 51.9% in our data. Scores in this dataset were however better than a study in Brazil, which found 91% of adolescents had a ‘poor’ diet, using a locally modified version of the HEI (52). This reflects the marked impact of socioeconomic and cultural factors on diet among young people, making local studies critical to understanding and planning interventions.

The component-level HEI findings across the adequacy and moderation domains of the HEI provide insight into potential targeted interventions at the local level. In this study, low HEI scores were primarily driven by high consumption of refined grains and added sugars, alongside consistently poor intake of key food groups such as fruits, vegetables, legumes, and plant-based proteins. These gaps reflect limited dietary diversity and quality, which likely contributed to the inverse association observed between diet quality and obesity risk. Similar patterns have been reported in the Eastern Mediterranean Region (EMR), where rapid social and economic changes have led to diets characterized by low intake of nutrient-dense foods and a growing reliance on energy-dense, nutrient-poor products (53). Additionally, early dietary exposures may play a compounding role. Thompson et al. (2021) demonstrated that earlier introduction of sugar-sweetened beverages in the first 2 years of life was significantly associated with lower HEI scores at age three, with negative impacts seen across added sugar, whole grain, and fruit components (54). These findings suggest that both early life feeding practices and ongoing food environment exposures shape long-term dietary patterns. This means that targeted policy around limiting added sugars, and promotion of the use of whole grains where possible could have significant impacts on adolescent diet quality. Policy responses in food labeling requirements mandated maximum limits of harmful food components in packaged foods, and subsidies for whole grain foods could lead to effective national scale improvement of youth diet. In addition, public education on the importance of whole grains and the detrimental effects of added sugars targeted at parents and adolescents could improve consumer awareness. Lack of wholegrain foods was also identified in the adequacy analyses, along with low vegetable consumption, as an area in which improvements could be made. These areas could likewise be targeted for public education, such as has been successfully undertaken in Denmark in recent years (55). Importantly, any policy responses should be targeted at both adolescents and caregivers, with key messaging tailored to both for maximum effect. Moreover, although our analysis identified a statistically significant association between skipping breakfast and obesity risk, this finding should be interpreted with caution due to the very small number of children in the sample who reported not eating breakfast.

The difference in physical activity levels between obese and non-obese children in this study highlights a critical behavioral factor associated with childhood obesity. It reflects a broader regional concern regarding insufficient physical activity among youth. A meta-analysis conducted in the UAE revealed that approximately one quarter of the adolescent population leads a completely sedentary lifestyle, while only a minority engages in moderate or vigorous physical activity, with higher activity levels more common among male adolescents (56). Similar findings have been reported in Qatar, where the majority of schoolchildren were found to spend most of their school day in sedentary pursuits, and only 39 percent met the recommended 30 min of moderate-to-vigorous physical activity during school hours (57). Notably, girls aged 9 years exhibited significantly lower activity levels compared to boys of the same age group (57). Although several school-based interventions in the Middle East countries have demonstrated improvements in physical activity levels, most programs were multi-component and not specifically designed to address physical activity in isolation, limiting their effectiveness and sustainability (58). Therefore, interventions must account for gender-specific barriers, social norms, and environmental constraints to close the activity gap between weight categories and support long-term health outcomes.

While this is the largest study of its kind in the Middle East, our results have some important limitations that must be considered. Most importantly, this is a secondary analysis of a cross-sectional data set, and as such, no causative relationships can be inferred. Secondly, this study was conducted across the three most populous emirates in the UAE, all of which are heavily urbanized, and have higher SES than some other regions. Policymakers and healthcare providers need to consider the unique circumstances of people outside the major urban centers, where food scarcity and access, as well as lower health literacy, and different food practices, may have major influences.

### 4.1 Clinical implications and recommendations

The high proportion of children with poor overall diet quality highlights the need for routine diet-quality screening in pediatric visits, with brief counseling and follow-up where feasible. Given the inverse association between higher HEI and overweight/obesity, clinicians could prioritize component-focused counseling that addresses the most important gaps observed in our cohort such as insufficient intake of vegetables, whole grains, seafood, and plant proteins, alongside with excess intake of refined grains and added sugars. This could be addressed using simple swaps and culturally appropriate meal recommendations. Children of employed mothers were less likely to achieve moderate-to-good HEI; therefore, nutrition counseling could focus on time-saving strategies for busy households. Moreover, clinicians should reinforce physical-activity guidelines. Finally, while breakfast consumption showed a protective direction, the estimate is based on very small numbers and should be interpreted cautiously; clinicians may still emphasize regular, nutrient-dense breakfasts aligned with HEI components.

## 5 Conclusion

Children in the UAE broadly have poor diet quality, as assessed by the HEI, supporting the integration of brief diet-quality screening and counseling in pediatric and school health encounters. In addition, HEI scores were negatively associated with the risk of overweight and obesity and maternal employment, while protective associations were found with paternal education. Lack of wholegrain and vegetable intake, and excess added sugar and refined grains were the most important contributors to the HEI scores in the study. Given the increasing rates of both overweight and obesity, as well as cardiometabolic diseases in the UAE, urgent public health interventions are required to improve diets in early life. Interventions should prioritize increasing access to and consumption of whole foods through school meal reforms, community-based nutrition education, and policies that support healthy food availability at home. Additionally, aligning dietary strategies with efforts to increase weekday physical activity may yield additive benefits. Particular attention should be given to supporting working mothers through workplace nutrition initiatives and family-oriented guidance, while also leveraging paternal engagement as a potential vector for improving children’s dietary habits. Future work should prioritize longitudinal cohorts and pragmatic trials that pair diet-quality targets with feasible behavior-change supports and evaluate implementation in schools and communities. Addressing these determinants at both household and systemic levels will be critical to improving long-term health outcomes for the pediatric population in the UAE.

## Data Availability

The original contributions presented in the study are included in the article/supplementary material, further inquiries can be directed to the corresponding author.
